# Microarray Analysis of Small Extracellular Vesicle-Derived miRNAs Involved in Oxidative Stress of RPE Cells

**DOI:** 10.1155/2020/7658921

**Published:** 2020-10-27

**Authors:** Ke Mao, Xingwei Wu

**Affiliations:** ^1^Department of Ophthalmology, Renji Hospital, School of Medicine, Shanghai Jiao Tong University, Shanghai, China; ^2^Department of Ophthalmology, Shanghai General Hospital, National Clinical Research Center for Eye Diseases, Shanghai Key Laboratory of Ocular Fundus Diseases, Shanghai Engineering Center for Visual Science and Photomedicine, School of Medicine, Shanghai Jiao Tong University, Shanghai, China

## Abstract

The aim of this study was to investigate the miRNA profiles of nanosized small extracellular vesicles (sEVs) from human retinal pigment epithelial (RPE) cells under oxidative damage. ARPE-19 cells were cultured with ox-LDL (100 mg/L) or serum-free medium for 48 hours, sEVs were then extracted, and miRNA sequencing was conducted to identify the differentially expressed genes (DEGs) between the 2 groups. RNA sequence results were validated using quantitative real-time PCR. The Gene Ontology (GO) enrichment, Kyoto Encyclopedia of Genes and Genomes pathway, and ingenuity pathway analyses (IPA) were performed for the DEGs. Results revealed that oxidative stress inhibited RPE cell viability and promoted sEV secretion. A total of 877 DEGs from sEVs were identified, of which 272 were downregulated and 605 were upregulated. In total, 66 enriched GO terms showed that the 3 most significant enrichment terms were cellular processes (biological processes), cell (cellular component), and catalytic activity (molecular function). IPA were used to explore DEGs associated with oxidation damage and further construct a miRNA-target regulatory network. This study identified several DEGs from oxidation-stimulated RPE cells, which may act as potential RNA targets for prognosis and diagnosis of RPE degeneration.

## 1. Introduction

Oxidative damage is one of the major contributors to retinal degenerative diseases such as age-related macular degeneration (AMD) [1]. AMD is a multifactorial disease in which oxidative stress serves as a key component. The retinal pigment epithelium (RPE) is a highly specialized, polarized epithelium, which is in intimate contact with the outer segments of the photoreceptor and Bruch's membrane [2]. PRE cells are particularly metabolically active, highly oxygenated, and vulnerable to oxidative stress under exposure to photosensitizers such as lipofuscin [3]. Oxidative stress induces cell apoptosis through reactive oxygen species, thereby leading to RPE dysfunction [4].

Exosome refers to one form of extracellular nanometer-sized vesicle, which mediates multiple extra- and intercellular activities, including cell-cell communication, immune modulation, extracellular matrix turnover, stem cell division/differentiation, neovascularization, and cellular waste removal [5]. RPE cells secrete extracellular vesicles (EVs) in response to oxidative stress, resulting in angiogenesis in endothelial cells [6]. Exosomal biological markers CD63 and LAMP2 have been found in the drusen of AMD patients and stressed RPE cells, which suggests that the drusen is initiated by intracellular proteins from RPE cells and becomes extracellular via the exosome [7]. Small extracellular vesicles (sEVs) contain multiple functional molecules such as mRNA, microRNA (miRNA), and proteases. miRNAs are small noncoding RNA molecules, which inhibit several targeting mRNA expressions. Genetic mutations of miRNAs induce pathophysiological and immunological dysfunction in RPE cells. A related study suggested exosomal miRNA variations as predictive biomarkers in AMD disease [8]. Here, we aimed to screen for differentially expressed miRNA profiles in sEVs derived from oxidative stress-stimulated RPE cells and identify potential functional miRNAs, which may be associated with RPE oxidation.

## 2. Materials and Methods

### 2.1. Cell Culture and Oxidative Stress Induction

The human RPE cell line (ARPE-19) is transformed and maintained at 1 × 10^6^ cells/mL culture in DMEM/F12 medium (Gibco Life Technologies, Carlsbad, CA, USA) containing 10% fetal bovine serum (FBS; HyClone, Shanghai, China), penicillin/streptomycin (1 : 100, Sigma, USA), 4 mM glutamine, and 0.19% HEPES (Sigma), in a humidified incubator at 37°C and 5% CO_2_. Cells were seeded and grown to 70-80% confluence before being placed in a serum-free medium (SFM) for 24 hours, then randomized into SFM or human oxidized low-density lipoprotein (ox-LDL, 100 mg/L, AppliChem, Darmstadt, Germany) groups for 48 hours.

### 2.2. CCK8 Assay for RPE Cell Viability

ARPE-19 cells were seeded at a density of 1 × 10^4^ cells/100 *μ*L/well in 96-well plates. After the treatment mentioned above, 10 *μ*L of XTT (BBI Life Sciences, China) solution was added into each well for 1 hour at 37°C. Cell viability was determined by measuring absorbance at 450 nm using a microplate spectrophotometer (Molecular Devices, Sunnyvale, CA, USA).

### 2.3. Exosome Isolation and Transmission Electron Microscopy Imaging

Exosomes were isolated from ARPE-19 cells using multistep differential centrifugation [9]. ARPE-19 cells were centrifuged at 300 × g for 10 minutes at 4°C. Subsequently, the supernatant was subjected to the following centrifugation steps: 2000 × g for 10 minutes, 10,000 × g for 30 minutes, and 100,000 × g for 70 minutes. The resulting sEVs were finally resuspended in PBS and centrifuged at 100,000×g for 70 minutes again. The morphology of sEVs was visualized using the Hitachi transmission electron microscope operated at 80 kV (Hitachi, Japan).

### 2.4. Western Blot Analysis

As described previously [10], after incubating for 5 min at 90°C with loading buffer (Life Technologies, Australia), 10 *μ*g of exosomes in each group was electrophoresed on NuPAGE Novex 4–12% Bis-Tris Gels (Life Technologies, USA). Gels were transferred onto PVDF membranes using the Trans-Blot Turbo system. Membranes were blocked in 2% BSA solution for 3 hours and then probed overnight with primary exosomal marker protein antibodies: anti-Hsp70 (ab134045, Abcam, Cambridge, UK), anti-CD63 (ab181606, Abcam), and anti-TSG101 (ab125011, Abcam) at 4°C, followed by incubation with a secondary antibody for 3 hours. The ChemiDoc XRS gel documentation system (Bio-Rad Laboratories, USA) was used to quantify the immune-reactive proteins, and *β*-actin was used as a loading control for each lane. Each indicated band was quantified and normalized to *β*-actin through ImageJ software.

### 2.5. miRNA Extraction and miRNA Sequencing

As reported previously [11], RNA extraction was performed using the Total Exosome RNA and Protein Isolation Kit (catalog # 4478545; Invitrogen, USA) according to the provided instructions. 200 ng-1 *μ*g RNA in final volume of 30 *μ*L solution was collected for each sample. Total RNA quantity and quality (260/280 absorbance ratio) were assessed using NanoDrop 2000 (Thermo Fisher Scientific, Waltham, MA, USA) and Agilent 2100 Bioanalyzer to test concentration and inorganic ions or polycarbonate contamination. miRNA sequence was isolated by BGI Company (China) based on previous instructions [12]. cDNA libraries were constructed using the Ion Total RNA-Seqv2 kit (Life Technologies, USA) (*n* = 3 for each group) and purified using AMPure beads (Beckman Coulter). Emulsion PCR and enrichment of cDNA-conjugated particles were performed with an Ion OneTouch 200 Template Kit v2 DL (Life Technologies). The final cDNA samples were sequenced single end on the HiSeq 2000 System with a 50 bp read length.

### 2.6. Bioinformatics Analysis of the Data

Raw data was filtered to eliminate low-quality reads, primers, adaptors, and other contaminants. Following this, we summarized the length distribution and common and specific sequences between samples. After filtering, the remaining tags were called clean tags and stored in FASTQ format. Bowtie2 was used to map clean reads to the reference genome and other sRNA databases. To identify differentially expressed genes (DEGs), differentially expressed miRNAs (DEMIs) were screened out using the limma package through the thresholds of fold change > 2 or <0.5 and adjusted *p* value of <0.05 [13].

To perform Gene Ontology (GO) enrichment analysis, we mapped all genes to GO terms in the database, which calculated the gene numbers for every term. The hypergeometric test was then used to find significantly enriched GO terms in the input gene list.

The Kyoto Encyclopedia of Genes and Genomes (KEGG) pathway was used to perform pathway enrichment analysis. This analysis identified significantly enriched metabolic or signal transduction pathways from target genes of DEGs when compared with the whole genome background. The *p* value was corrected using the Bonferroni method; a corrected *p* value < 0.05 was considered significant.

In this study, experimentally verified miRNA-mRNA regulatory pairs were obtained using TargetScan and miRanda and a miRNA-target regulatory network was constructed by comparing the DEGs with obtained miRNA-gene regulatory pairs.

### 2.7. Validation of miRNA Expression Using Quantitative Real-Time PCR (RT-PCR)

In order to validate initial miRNA sequence results, the 10 most significant up- or downregulated miRNAs were selected for further RT-PCR tests as reported previously [14]. Total RNA was isolated using TRIzol reagent and the quality and quantity of RNA was measured using a NanoDrop 2000 spectrophotometer. Each reverse transcription reaction mixture contained 10 mL of SYBR Green Master Mix, 0.5 mL of miR-RT primers F (10 mM), 0.5 mL of miR-RT primers R (10 mM), and RNase-free H_2_O. The RT-PCR reactions for the selected 10 miRNAs were performed using the ViiA 7 Real-Time PCR System (ABI, USA) under the following conditions: 95°C for 1 min, followed by 40 PCR cycles (95°C for 10 s and then 60°C for 20 s). miRNA expression was normalized to the endogenous reference gene *GAPDH*. Each sample was analyzed in triplicate. Specific primers were produced by BIOTNT Company (Shanghai, China). Relative quantification was achieved by the comparative 2^−*ΔΔ*ct^ method.

### 2.8. Statistics

The data were analyzed with a one-way analysis of variance (ANOVA) using the statistical program SPSS 17.0. All data were presented as mean ± SD. *p* value < 0.05 was considered statistically significant.

## 3. Results

### 3.1. ox-LDL Decreases ARPE-19 Cell Viability

**Figure 1:**
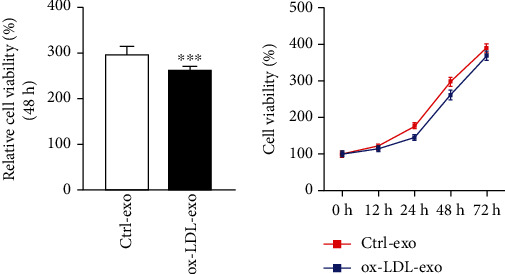
ox-LDL reduced RPE cell viability. ARPE-19 cells were treated with control (serum-free medium) or ox-LDL (100 mg/L) for 48 hours. Cell viability was tested by CCK8 assay. Data are expressed as mean ± SD (*n* = 3). Experiments were repeated 3 times. ^∗∗∗^*p* < 0.001 vs. the control group.

We first measured the cytotoxicity of ox-LDL to ARPE-19 cells after 48 hours.  Figure 1 shows that cell viability in the ox-LDL group was significantly lower than that in the control group (*p* < 0.05), which indicated its cytotoxicity and aligned with previous conclusions [15, 16].

### 3.2. Characterization of sEVs and Biological Marker Protein Detection

**Figure 2:**
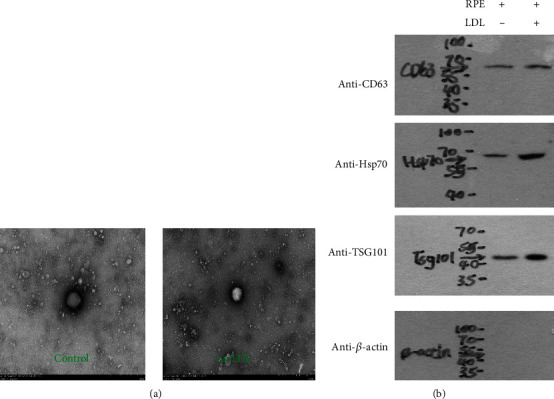
Transmission electron microscopy images of exosome in the ox-LDL and control groups (a) and western blot results of exosomal marker proteins (b).

Transmission electron microscopy images of sEVs derived from both groups revealed the presence of distinct vesicles with an average diameter of 106 ± 7.62 nm (Figure 2(a)). The vesicles were also positive for exosomal markers. CD63 is the general tetraspanin protein used as the exosomal “star marker” [17]. TSG101 and Hsp70 are also commonly used for exosome detection [18, 19]. As shown in Figure 2(b), we found that the expression levels of TSG101 and Hsp70 were statistically higher in the ox-LDL group than the control, but it did not reach a significant difference (*p* > 0.05).

### 3.3. Differential Expression of miRNA Profiles in sEVs Isolated from the Oxidative and Control RPE Cells

**Figure 3:**
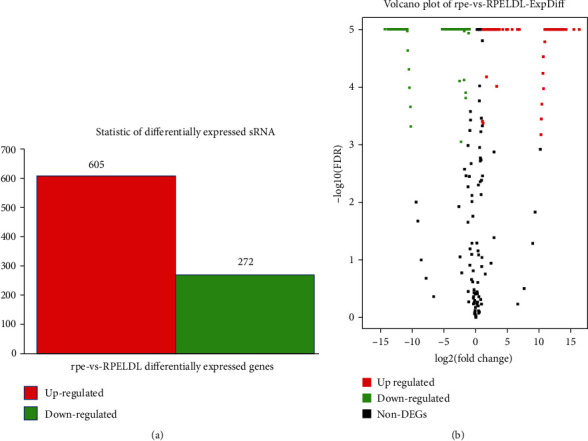
General RNA-Seq analysis of sEV-derived miRNAs and statistical analysis of differentially expressed genes between the ox-LDL and control groups. (a) Histogram of DEGs between 2 groups. (b) Volcano plot of DEGs. *p* value < 0.05 was considered significant.

In order to identify the influence of oxidation on miRNA profiles from sEVs of RPE cells, miRNA sequence was sequenced after treatment for 48 hours.  Figure 3 shows that 877 significantly differentially expressed miRNAs had been screened between the ox-LDL and control groups, among which 272 were downregulated and 605 were upregulated. The top 10 differentially expressed genes are listed in Table 1.

### 3.4. DEG Validation Using RT-PCR

**Figure 4:**
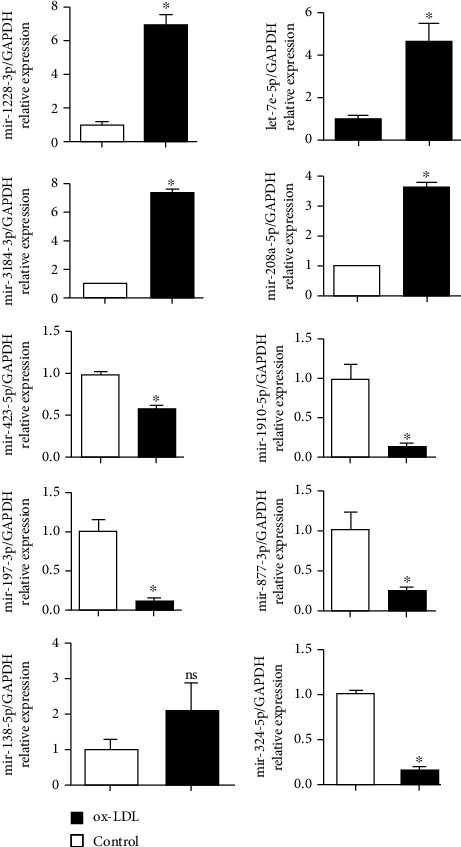
Validation of the top 10 selected DEGs screened from miRNA sequence by qRT-PCR tests. ARPE-19 cells were treated as before, and miRNAs were extracted from sEVs. Data was expressed as mean ± SD (*n* = 6). Experiments were repeated three times. ^∗^*p* < 0.05 vs. the control group. ns: no significance.

The top 10 selected DEGs were further validated using RT-PCR (Figure 4). PCR tests revealed similar results of RNA sequence, except that miR-138-5p showed insignificant differences between the two groups (*p* > 0.05).

### 3.5. Gene Ontology Enrichment Analysis

**Figure 5:**
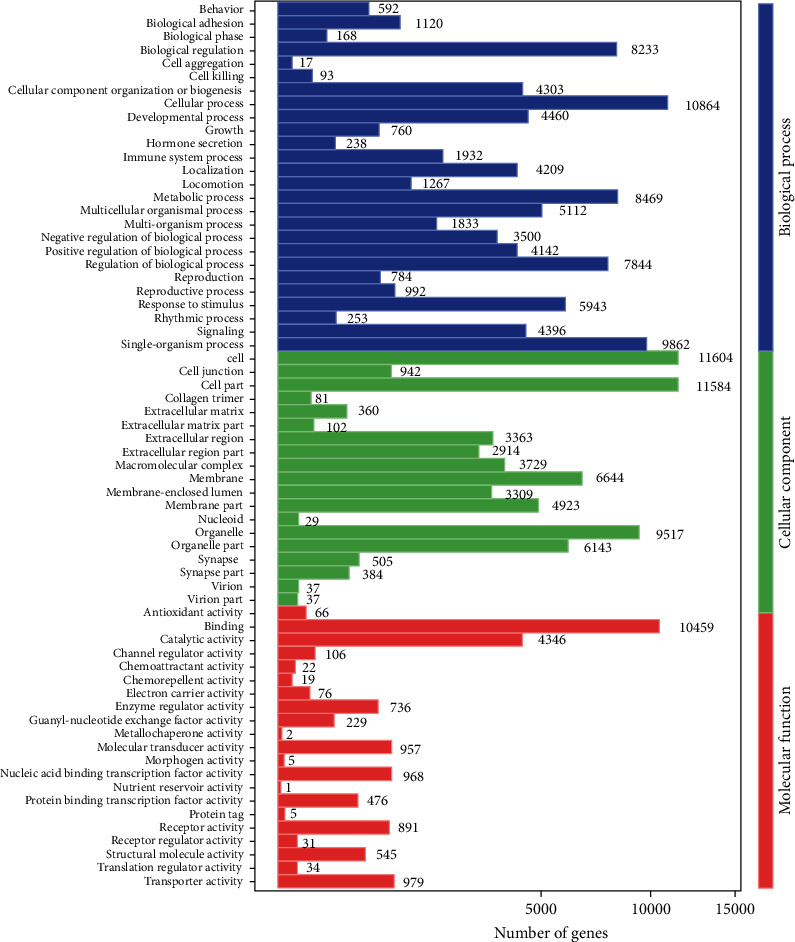
Go enrichment analysis of DEGs between 2 groups. Blue, green, and red bars represent the enrichment and numbers of DEGs in the biological process, cellular component, and molecular function, respectively.

The GO analysis contains three ontologies: biological processes, molecular function, and cellular components. We identified 66 enriched GO terms, among which 26 belong to biological processes, 21 belong to molecular function, and 19 belong to cellular components. The three most enriched biological process terms were cellular processes, single-organism processes, and metabolic processes. Meanwhile, cell, cell part, organelle, catalytic activity, transporter activity, and transporter activity were the most enriched GO terms of cellular components and molecular function, respectively (Figure 5).

### 3.6. KEGG Pathway Enrichment Analysis

**Figure 6:**
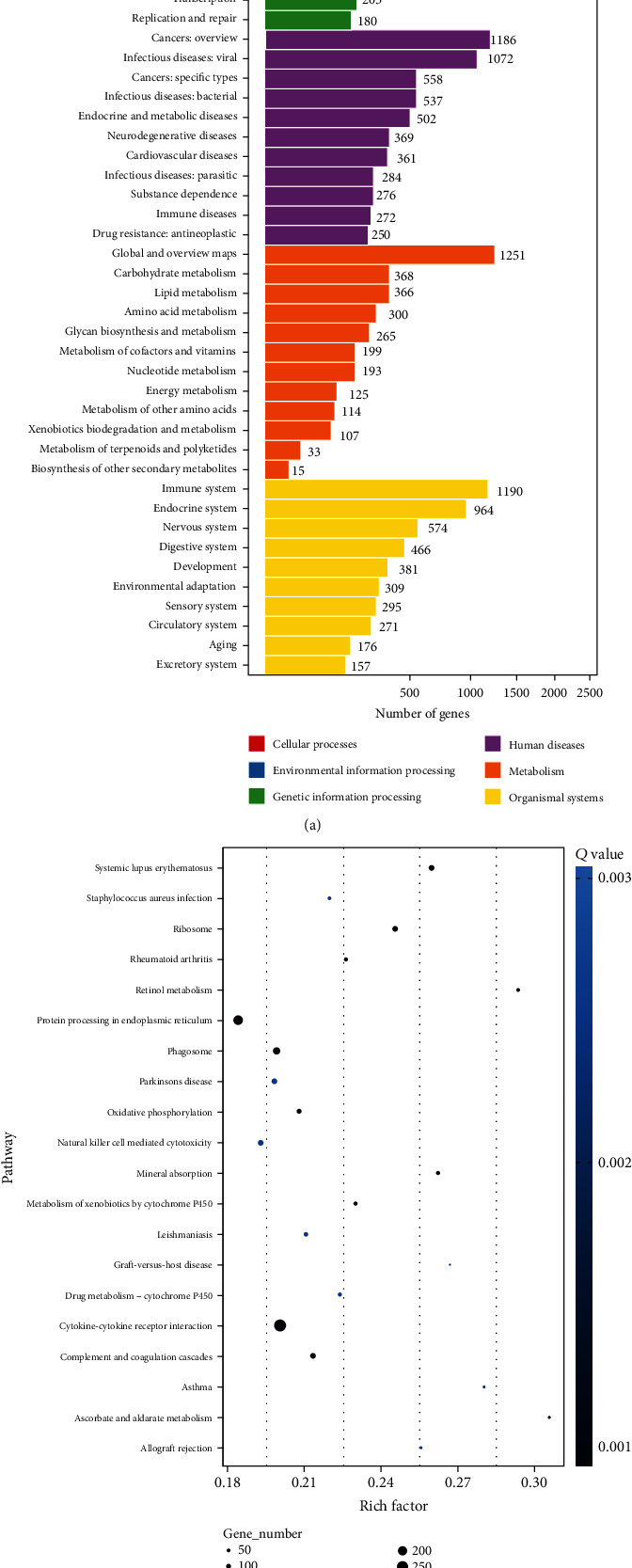
KEGG pathways analysis of DEGs between 2 groups. (a) 6 categories of biological functions and numbers of genes in different pathways. (b) Rich factors of the 20 most enriched pathways. The sizes of circles correspond to gene numbers. The colors correspond to the *Q* value.

KEGG analysis classified DEGs into 6 categories according to their biological functions: cellular processes (4 pathways), environmental information processing (3 pathways), genetic information processing (4 pathways), human diseases (11 pathways), metabolism (12 pathways), and organismal systems (10 pathways). The 20 most enriched pathways are presented in Figure 6.

### 3.7. Functional Exploration with Ingenuity Pathway Analysis

Based on the KEGG and Gene Ontology results, we further searched for related functional genes and associated pathways by ingenuity pathway analysis (IPA) from DEGs. We identified several pathways and genes which related to AMD (6 pathways), lipid metabolism (4 pathways), oxidative damage (5 GO terms), cellular inflammation (5 GO terms), and choroidal neovascularization (GO:0045765) (Table 2).

### 3.8. miRNA-Target Regulatory Network Analysis

**Figure 7:**
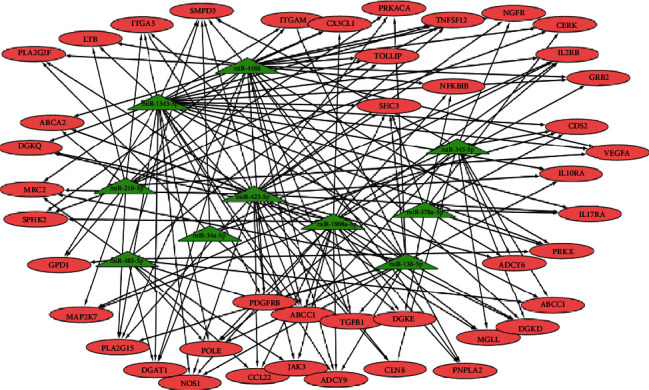
microRNA-target regulatory networks of differentially expressed genes (DEGs). Green triangles represent miRNAs; red circles represent targeting genes.

We used TargetScan and miRanda software to predict possible targeted mRNAs for DEGs and associated their intersections with the IPA results; a total of 10 miRNAs and 43 targeted mRNAs formed a miRNA-target regulatory network (Figure 7).

## 4. Discussion

Oxidative stress has been recognized as a major influence in AMD pathophysiology, and RPE appears to be the main site of damage [2]. Oxidative damage of the RPE layer originates from the digestion of photoreceptor outer segments and other reactive oxygen species. RPE damage occurs in multiple locations within the central part of the eye and finally forms a region of atrophy by the bystander effect, which is mediated via EVs [20]. In this research, ox-LDL decreased ARPE-19 cell viability and promoted sEV secretion. RNA sequences and RT-PCR tests confirmed a downregulation of *miR*-*1910*-*5p* in sEVs of the ox-LDL group, which is contrary to a similar study that found that H_2_O_2_ increases *miR*-*1910*-*5p* concentrations in ARPE-19 cells [21]. *miR*-*324*-*5p* was reported to be expressed in plasma of wet AMD patients, and we found a decreased expression in the ox-LDL group [22]. Desjarlais et al. [23] demonstrated an upregulation of *let*-*7g*-*5p* (>570%) in oxygen-induced retinopathy models during the neovascularization phase, which is consistent with our result. Other authors reported significant changes of *miR*-*192*, *let*-*7c*, *miR*-*183*, *miR*-*27a*, *miR*-*27b*, *miR*-*361*-*5p*, *miR*-*335*, and *miR*-*30c* in experimental AMD models, which were also observed in our study (see Table 3) [24–26]. KEGG analysis suggested cytokine-cytokine receptor interactions and phagosome and protein processing in the endoplasmic reticulum to be the most significant enrichment items. In agreement with other reports, cancer-related pathways are also involved in DEGs of sEVs in our study [15]. Further research is needed to explore the specific roles of these pathways.

IPA screened out 6 key pathways related to AMD and 4 GO terms related to oxidative damage. *miR*-*138*-*5p*, *miR*-*345*-*5p*, *miR*-*210*-*5p*, *miR*-*34a*-*5p*, *miR*-*1908*-*5p*, *miR*-*1343*-*3p*, *miR*-*485*-*5p*, *miR*-*423*-*5p*, and *miR*-*4488* are associated with oxidative stress and AMD.

The miRNA-target regulatory network consists of several miRNAs and predicted targeting mRNAs. *miR*-*1343* was proved to be activated in response to stress in epithelial cells and targets both TGF-*β* receptors, which in turn contribute to the progression of angiogenesis in wet AMD [27, 28]. *miR*-*4488* was demonstrated to be involved in sphingolipid signaling and to modulate endoplasmic reticulum stress marker PERK in ARPE-19 cells [29]. *miR*-*345*-*5p* was found to be downregulated in ARPE-19 cells undergoing oxidative stress, which is also consistent with our findings [21]. A *miR*-*210*-*5p* variant was demonstrated to affect *CFB* expression in RPE cells and modulate the *CFB* level in AMD patients [30]. *miR*-*423*-*5p* is significantly increased in the proliferative diabetic retinopathy eyes and believed to modulate angiogenic signals [31]. In this study, it was downregulated Iafter ox-LDL treatment (FC = −14.52, *p* < 0.05). *miR*-*1908*-*5p* plays an important role in regulating lipid metabolism in blood, and *miR*-*378a*-*5p*/*138*-*5p*/*34a*-*5p* are important miRNAs mediating lipid metabolism, tumor angiogenesis, and oxidative stress [32–36]. According to the IPA results, miRNA-target mRNA network, and previous references, *miR*-*138*-*5p*, *miR*-*345*-*5p*, *miR*-*210*-*5p*, *miR*-*34a*-*5p*, *miR*-*1908*-*5p*, *miR*-*1343*-*3p*, *miR*-*485*-*5p*, *miR*-*423*-*5p*, and *miR*-*4488* may serve as potential RNA targets for prognosis and diagnosis of RPE degeneration.

Compared with previous attempts at this type of analysis, a lower number of identified DEGs coincided with this study, which is probably due to the use of different oxidative injury models in RPE cells. Our research investigated acute responses of RPE cells to oxidative stress, which could not represent pathogenesis of AMD since it is a long-term effect.

## 5. Conclusion

n conclusion, exploring oxidative stress-induced miRNA profiles has led us to potential prospects in evaluating RNA variation in sEVs, which may be useful as prognostic and diagnostic tools in the future.

## Figures and Tables

**Table 1:** List of the top 10 DEGs between the ox-LDL and control groups.

Gene ID	log2Ratio (RPELDL/rpe)	*p* value	FDR	Description	Primer sequence (5′ → 3′)
hsa-miR-3184-3p	16.27193714	<0.001	<0.001	Up	TCCTCTTCTCCCTCCTCCCA
hsa-let-7e-5p	15.46467337	<0.001	<0.001	Up	AGCTGGTGTTGTGAATCAGG
hsa-miR-208a-5p	13.98992663	<0.001	<0.001	Up	CGCATCCCCTAGGGCATTGG
hsa-miR-138-5p	13.57222651	<0.001	<0.001	Up	TAGTGCAATATTGCTTATAG
hsa-miR-1228-3p	13.13089227	<0.001	<0.001	Up	AAAGTCTCGCTCTCTGCCCC
hsa-miR-423-5p	-14.52833201	<0.001	<0.001	Down	GGAGCGAGATCCCTCCAAAAT
hsa-miR-1910-5p	-13.02410078	<0.001	<0.001	Down	GAGCTTTTGGCCCGGGTTAT
hsa-miR-197-3p	-11.56985561	<0.001	<0.001	Down	GGCTGTTGTCATACTTCTCATGG
hsa-miR-877-3p	-10.27612441	<0.001	<0.001	Down	TCACAGTGGCTAAGTTCTGC
hsa-miR-324-5p	-9.409390936	<0.001	<0.001	Down	TGAGGGGCAGAGAGCGAG

FDR: false discovery rate.

**Table 2:** List of DEGs in IPA.

Function	Pathway or GOID	Name (Homo sapiens (human))	Count	Gene ID
AMD	hsa02010	ABC transporters	8	miR-345-5p, miR-210-5p, miR-34a-5p, miR-1908-5p, miR-485-5p, miR-1343-3p, miR-423-5p, miR-4488
	hsa03420	Nucleotide excision repair	5	miR-138-5p, miR-345-5p, miR-1908-5p, miR-1343-3p, miR-485-5p
	hsa04060	Cytokine-cytokine receptor interaction	12	miR-138-5p, miR-345-5p, miR-210-5p, miR-378a-5p, miR-34a-5p, miR-1908-5p, miR-1343-3p, miR-485-5p, miR-423-5p, miR-4488, miR-210-5p, miR-423-5p
	hsa04062	Chemokine signaling pathway	9	miR-138-5p, miR-345-5p, miR-210-5p, miR-378a-5p, miR-1908-5p, miR-1343-3p, miR-485-5p, miR-423-5p, miR-4488
	hsa04145	Phagosome	7	miR-138-5p, miR-210-5p, miR-1908-5p, miR-1343-3p, miR-485-5p, miR-423-5p, miR-4488
	hsa04620	Toll-like receptor signaling pathway	6	miR-345-5p, miR-210-5p, miR-1908-5p, miR-1343-3p, miR-485-5p, miR-4488
Lipid metabolism	hsa00561	Glycerolipid metabolism	10	miR-138-5p, miR-345-5p, miR-378a-5p, miR-34a-5p, miR-1908-5p, miR-1343-3p, miR-485-5p, miR-423-5p, miR-4488, miR-210-5p
	hsa00564	Glycerophospholipid metabolism	10	miR-138-5p, miR-345-5p, miR-210-5p, miR-378a-5p, miR-34a-5p, miR-1908-5p, miR-1343-3p, miR-485-5p, miR-423-5p, miR-4488
	hsa00565	Ether lipid metabolism	3	miR-34a-5p, miR-423-5p, miR-4488
	hsa00600	Sphingolipid metabolism	6	miR-34a-5p, miR-1908-5p, miR-1343-3p, miR-485-5p, miR-423-5p, miR-4488
Oxidative damage	GO:1902175	Regulation of oxidative stress-induced intrinsic apoptotic signaling pathway	0	
	GO:1900407	Regulation of cellular response to oxidative stress	9	miR-138-5p, miR-345-5p, miR-210-5p, miR-34a-5p, miR-1908-5p, miR-1343-3p, miR-485-5p, miR-423-5p, miR-4488
	GO:0001306	Age-dependent response to oxidative stress	9	miR-138-5p, miR-345-5p, miR-210-5p, miR-34a-5p, miR-1908-5p, miR-1343-3p, miR-485-5p, miR-423-5p, miR-4488
	GO:0036473	Cell death in response to oxidative stress	4	miR-138-5p, miR-210-5p, miR-1343-3p, miR-4488
	GO:1902882	Regulation of response to oxidative stress	9	miR-138-5p, miR-345-5p, miR-210-5p, miR-34a-5p, miR-1908-5p, miR-1343-3p, miR-485-5p, miR-423-5p, miR-4488
Cellular inflammation	GO:0002532	Production of molecular mediator involved in inflammatory response	8	miR-4488, miR-345-5p, miR-378a-5p, miR-34a-5p, miR-1908-5p, miR-1343-3p, miR-485-5p, miR-423-5p
	GO:0002534	Cytokine production involved in inflammatory response	8	miR-4488, miR-345-5p, miR-378a-5p, miR-34a-5p, miR-1908-5p, miR-1343-3p, miR-485-5p, miR-423-5p
	GO:0002537	Nitric oxide production involved in inflammatory response	0	
	GO:0002540	Leukotriene production involved in inflammatory response	0	
	GO:0002541	Activation of plasma proteins involved in acute inflammatory response	0	
Choroid angiogenesis	GO:0045765	Regulation of angiogenesis	7	miR-4488, miR-138-5p, miR-345-5p, miR-210-5p, miR-1908-5p, miR-1343-3p, miR-423-5p

**Table 3:** List of DEGs related to AMD in previous studies.

Gene ID	log2Ratio (RPELDL/rpe)	*p* value	FDR	Description
hsa-*miR*-*192* [12]	-10.98	<0.001	<0.001	Down
hsa-*let*-*7c* [25]	5.60	<0.001	<0.001	Up
hsa-*miR*-*183* [24]	12.16	<0.001	<0.001	Up
hsa-*miR*-*27a* [25, 26]	12.10	<0.001	<0.001	Up
hsa-*miR*-*27b* [22]	4.99	<0.001	<0.001	Up
hsa-*miR*-*361*-*5p* [12]	-11.94	<0.001	<0.001	Down
hsa-*miR*-*335* [12, 22]	-12.06	<0.001	<0.001	Down
hsa-*miR*-*30c* [25]	10.51	<0.001	<0.001	Up

## Data Availability

The data that support the findings of this study are available from the corresponding author upon reasonable request.

## Abbreviations

sEVs: Small extracellular vesicles
RPE: Retinal pigment epithelium
ox-LDL: Oxidized low-density lipoprotein
SFM: Serum-free medium
DEGs: Differentially expressed genes
GO: Gene Ontology
KEGG: Kyoto Encyclopedia of Genes and Genomes
IPA: Ingenuity pathway analyses
AMD: Age-related macular degeneration.
